# Aetiology of fever among under fives in Lagos, Nigeria

**DOI:** 10.1186/1471-2334-14-S2-P42

**Published:** 2014-05-23

**Authors:** VNV Enya, N Idika, AG Mafe, KN Akinside, SI Smith, PU Agomo, IN Ibeh, SNC Wemambu

**Affiliations:** 1 Nigerian Institute of Medical Research, Lagos, Nigeria

## Introduction

Fever is a common presentation of HIV/AIDS, malaria and acute respiratory tract infections (ARIs) all of which are major causes of childhood morbidity and mortality in sub-Saharan Africa. Correct management requires accurate diagnosis of the cause of fever. This study was carried out to determine prevalence and pattern of in HIV, malaria and ARI in febrile children under 5 years of age.

## Materials and methods

A cross sectional study of all consenting and consecutive children 6- 59 months and their mothers / care-givers seen at the out patient department of Massey Street Children’s, Hospital presenting with fever. Semi structured questionnaire was used to obtain information from the mothers / care-givers and laboratory investigations were done for febrile children using 5mls of venous blood aseptically collected for blood culture, HIV, PCV and Malaria. Plasmids mediated antimicrobial resistance among bacteria isolates was determined. Data obtained from questionnaire, clinical and laboratory diagnoses were analyzed using EPI-INFO version 3.5.1 software.

## Results

Among 202 patients studied, 186 (92.1%) and 16 (7.9%) were clinically diagnosed as Malaria and ARIs respectively, but laboratory investigations showed 47 (23.3%) positive for malaria, ARIs 31(15.3%), HIV 10 (5.0%) and anaemia 12(5.9%). The results revealed the prevalence of HIV and ARIs co-infection as 10 (5%); HIV and Malaria 10(5%); Malaria and ARIs 27 (13.4%). The entire Malaria positive cases 47 (100%) were due to Plasmodium falciparum. Haemophilus influenzae isolates 5 (100%) and Streptococcus pneumoniae 23 (82.1%) isolates harboured plasmids which conferred multiple antimicrobial resistant on them.

## Conclusion

Fever in children may be as a result of Infection with HIV/AIDS, Malaria, ARI, or co-infection of any of the two diseases. Home management of fever was very high among mothers / care-givers. The commonest drugs used for treatment of childhood fever by mothers / care-givers were chloroquine and cotrimoxazole. Streptococcus pneumoniae the major organism isolated was highly resistant to cotrimoxazole.

